# Editorial: The role of probiotics, postbiotics, and microbial metabolites in preventing and treating chronic diseases, volume II

**DOI:** 10.3389/fcimb.2024.1442855

**Published:** 2024-06-28

**Authors:** Chuanlin Luo, Shengjie Li, Tingtao Chen

**Affiliations:** ^1^ Institute of Translational Medicine, Jiangxi Medical College, Nanchang University, Nanchang, China; ^2^ School of Pharmacy, Jiangxi Medical College, Nanchang University, Nanchang, China

**Keywords:** gut microbiota, chronic disease, probiotics, fecal microbiota transplantation, gut-X axis

Chronic diseases, such as cancers, cardiovascular disorders, diabetes, and chronic respiratory conditions, have become significant threats to human health. Almost all individuals suffering from chronic diseases require appropriate drug therapy and ongoing medical care during the rest of their life to achieve better clinical outcomes and quality of life, placing a significant therapeutic burden on patients. Additionally, high-impact essential interventions based on the insightful understanding of chronic diseases can be another critical way to control these conditions. The gut microbiota, a microbial community living in the gut, has been shown to greatly impact host health through direct and indirect pathways, i.e., the “gut-X” axis (Afzaal et al., 2022). Growing evidence suggests that the gut microbiota can be considered as an effective target for preventing and treating human diseases, including chronic conditions (Vijay and Valdes, 2022).

Currently, the gut microbiome translational research is of great interest. Considerable efforts are focused on manipulating the gut microbiota to treat and prevent diseases in preclinical and clinical studies. Several intervention approaches on the gut microbiota, including probiotics, prebiotics, and postbiotics, have been widely reported as next-generation therapeutics to restore the perturbed gut microbiota to health status and thereby treat and control diseases (Ji et al., 2023). This Research Topic expands seven publications for further exploring the role of probiotics, postbiotics, and microbial metabolites in preventing and treating chronic diseases (Xu et al., 2023).

Fecal microbiota transplantation (FMT) is a promising means for the treatment of diseases related to gut microbiota disorders (Smits et al., 2013). Gut microbiota dysbiosis is found to be related to traumatic brain injury (TBI). Is FMT effective in improving TBI? Hu et al. treated the TBI mice model with fecal microbiota collected from fresh healthy mice. After FMT treatment, neuronal damage in the mice was significantly alleviated, and the synaptic damage was improved. Meanwhile, the pathological damage of the intestinal tract and gut microbiota disorder in TBI mice were also improved. Their research verified by mice model that the FMT has an anti-neuroinflammatory effect on TBI and enclosed its mechanism which was related to the gut-brain axis and the modulation of peripheral immune cells.

Besides FMT, fecal bacteria-free filtrate transplantation (FFT) is also effective in regulating gut microbiota composition, thus to alleviate relevant disease symptoms. Zhang et al. treated a radiation exposure mouse model with FFT and then assessed the effectiveness. They reported that the FFT treatment reduced radiation-induced toxicity, together with a significant shift in the gut microbiome. After FFT treatment, the members of *Lachnospiraceae* family that have been reported to prevent radiation injury, termed as radioprotective microorganisms, showed a significant enrichment in the gut microbiota of recipients. Metabolome data showed that the levels of short-chain fatty acids (SCFAs) produced by intestinal microorganisms in FFT mice were also significantly increased. Their research indicates FFT is effective and safe for the treatment of radiation intestinal injury.

*Lactobacillus reuteri* is a probiotic strain that possesses multiple health benefits. Peng et al. summarized the effects *L. reuteri* strains on treating and preventing digestive system diseases. *L. reuteri* can be used to treat infantile colic and as an adjuvant treatment for diarrhea, constipation, and *Helicobacter pylori* infection. Besides, they summarized the mechanisms by which *L. reuteri* alleviates digestive system diseases. Their review provides valuable guidance for the clinical application of *L. reuteri*, although further study is still needed.

Research on Traditional Chinese Medicine (TCM) has demonstrated its effectiveness against cancer. Tripterygium glycosides (GTW), a TCM prescription, has been confirmed to enhance the effectiveness of cisplatin (DDP) chemotherapy against epithelial ovarian cancer (EOC); however, its mechanism remains to be studied. Zhan et al. aimed to explore this mechanism from the perspective of the gut microbiota. The results showed that the combination treatment of GTW and DDP significantly restored the relative abundance of *Lactobacillus* in mice. *L. acidophilus* transplantation or FMT with healthy mice verified the association of the anticancer effect of GTW and gut microbiota. This study indicates that GTW can effectively increase the chemosensitivity of DDP and repair the damaged intestinal barrier by restoring the balance of gut microbiota.

Two papers used Mendelian randomized (MR) analysis to study the relationship of gut microbiota with chronic conditions. A systematic review by Ren et al. focused on the genetic liability of gut microbiota for idiopathic pulmonary fibrosis (IPF) and lung function. They designed a bidirectional two-sample MR study, utilizing summary-level data from respective genome-wide association studies (GWAS) involving 211 gut microbial taxa, IPF, and lung function indicators (FEV1, FVC, and FEV1/FVC). They identified several taxa that were causally associated with IPF risk and lung function by multiple analyses. They also concluded that several lipid substances, including monounsaturated fatty acids, saturated fatty acids, and the ratio of omega-6 fatty acids to total fatty acids potentially contributed to mediating the genetic association between IPF and the gut microbiota, providing new strategies by dietary intervention with lipids to prevent IPF. Liu et al. analyzed the gut microbiota profiles among different obese types utilizing MR analysis. They found that *Ruminococcaceae UCG010* is an independent protective factor for obesity induced by excessive calorie intake. *Pasteurellaceae* and *Lactobacillus* are protective factors for localized adiposity and extreme obesity with alveolar hypoventilation, respectively. While *Butyricimonas* is an independent risk factor for medication-induced obesity. Additionally, they identified six gut microbiota that may cause different types of obesity. Their research provided novel insights for obesity treatment.

When conducting clinical and nutritional trials related to the gut microbiota, placebo effects in both healthy participants and patients should be considered. Pepoyan et al. aimed to assess the effects of a placebo on the gut microbiota composition in male Familial Mediterranean Fever (FMF) patients and to determine whether there is a significant diversity of bacteria independent of the placebo effect that can be directly utilized in clinical and nutritional trials. By conducting a partially randomized placebo trial including 15 FMF male patients and 15 healthy volunteers, and leveraging the high-density DNA microarray analysis, they found *Enterobacteriaceae* spp. could be defined as placebo-resistant among “healthy” gut bacteria, and for FMF male patients, *Akkermansia muciniphila* spp. shows relative resistance.

In summary, these publications highlight the specific role of gut microbiota on chronic conditions, including obesity, idiopathic pulmonary fibrosis, traumatic brain injury, digestive system diseases, radiation exposure, and cancer. These findings expand our knowledge of gut microbiome-host interactions and bring more effective approaches to modulate disturbed gut microbiota to treat and prevent chronic conditions. This Research Topic is valuable for the gut microbiota translational research on chronic diseases and ultimately promotes the development of probiotics-related industries.

## Author contributions

CL: Writing – review & editing, Writing – original draft. SL: Writing – review & editing, Writing – original draft, Funding acquisition. TC: Writing – review & editing, Supervision, Funding acquisition.
